# Cumulative IgG reagent carryover detected in carryover evaluation of urinary total protein testing using AU5800 biochemistry analyzer

**DOI:** 10.11613/BM.2025.020702

**Published:** 2025-04-15

**Authors:** Jialu Li, Guoxiang Bao

**Affiliations:** Clinical Laboratory Center, Shaoxing People’s Hospital, Shaoxing, Zhejiang, China

**Keywords:** carryover, urinary proteins, immunoglobulin G, laboratory testing

## Abstract

**Introduction:**

Accurate measurement of urinary total protein (UTP) is crucial for diagnosing renal and systemic diseases. This study aims to comprehensively evaluate potential carryover scenarios in UTP testing using AU5800 biochemistry analyzer and to identify factors influencing assay accuracy.

**Materials and methods:**

High-concentration quality control materials and pure water was used to evaluate specific sample probe carryover. Additionally, 24-hour mixed urine samples from patients were used to evaluate specific carryover from the reagent probe, mixbars, and cuvettes. For cumulative sample carryover evaluation, pure water was used as reagent and mixed serum as sample for continuous testing. During the process, 24-hour urine samples were interspersed, and UTP concentrations were measured at 0, 10, 20, and 30 minutes. Cumulative reagent carryover was evaluated by testing pure water sequentially with eight reagents sharing the same analytical unit and inner cuvettes as UTP, with UTP concentrations of 24-hour urine samples recorded at 0, 10, 20, and 30 minutes.

**Results:**

Specific carryover from the sample probe, reagent probe, mixbars, and cuvettes was not detected during carryover evaluation of UTP testing. However, a significant cumulative reagent carryover of Immunoglobulin G (IgG) reagent was observed, while no cumulative sample carryover was identified.

**Conclusions:**

The full range of possible carryover scenarios in UTP testing was evaluated using AU5800 biochemistry analyzer. Our data provide valuable references for evaluating carryover in laboratories, ensuring the accuracy and reliability of laboratory results.

## Graphical abstract

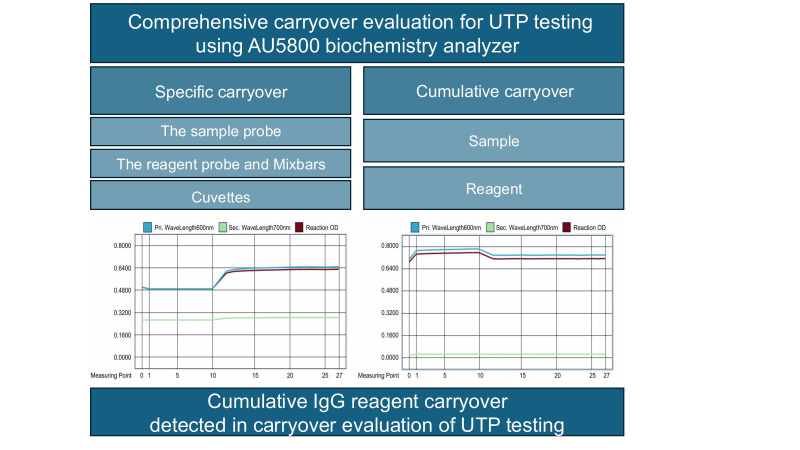

## Highlights

• Comprehensive carryover evaluation was conducted using AU5800 biochemistry analyzer

• No specific sample or reagent carryover was observed

• IgG reagent caused cumulative carryover, while cumulative sample carryover was absent

## Introduction

Carryover in clinical biochemistry analyzers has been extensively studied and is known to have a substantial impact on the precision and reliability of laboratory results (*1*, *2*). The rapid advancement of testing technology has increased the number of reagent positions on biochemical analyzers, leading to higher test volumes and greater potential for carryover, which is increasingly challenging to detect (*3*). This problem is potentially important for the accurate detection of urinary total protein (UTP). Urinary total protein is an early indicator of renal injury and plays a vital role in the early detection and management of chronic kidney disease (*4*). Accurate evaluation of UTP concentrations is essential for monitoring disease progression, evaluating treatment efficacy, and comprehending the influence of systemic diseases such as diabetes and hypertension on renal function (*5*).

AU5800 biochemistry analyzer is a high-throughput, fully automated clinical biochemistry analyzer widely used in clinical laboratories. It can perform up to 2000 tests *per* hour *per* analytical unit, with each unit equipped with 54 reagent positions (*6*). Currently, UTP in our laboratory is measured using the pyrogallol red-molybdate method. In this method, pyrogallol red combines with molybdic acid to form a red complex, which then binds with the protein under acidic conditions to produce a blue-purple complex, exhibiting a peak absorbance at 598 nm. The intensity of this blue-purple complex is proportional to the protein concentration present in the sample. Considering that the red complex can bind to all proteins, not just UTP, the significantly higher total protein concentration in serum samples compared to urine, the increased number of assay and assay combinations performed on the biochemical analyzer, and the presence of protein in certain reagent components, these factors collectively exacerbate the effect of carryover, posing significant concerns for the accuracy of UTP measurement (*7*, *8*).

Therefore, before UTP is routinely introduced as a clinical test, it is crucial to evaluate potential carryover in UTP detection to ensure accurate laboratory results. While most published articles focus on specific carryover evaluations without accurately simulating routine laboratory work, we aim to evaluate the full range of possible carryover scenarios in UTP testing using AU5800 biochemistry analyzer and to identify factors that could affect assay accuracy (*9*-*12*).

## Materials and methods

### Materials

The 24-hour urine and serum samples were obtained from leftover specimens of 20 patients undergoing clinical laboratory testing at Department of Clinical Laboratory Center, Shaoxing People’s Hospital, Shaoxing, China. To ensure homogeneity, the urine and serum samples from 20 patients were individually mixed prior to the experiments. The study was approved by the ethics committee of Shaoxing People’s hospital (Approval No.2024-092-02).

Clinical biochemical tests were performed on AU5800 biochemistry analyzer (Beckman Coulter, Brea, USA). Pure water was supplied by pure water system (TCHS-10ROE/1200KC, TianChuang Company, Hangzhou, China). Reagents for alkaline phosphatase (ALP), creatinine (Crea), cholinesterase (CHE), and rheumatoid factor (RF) were from Beckman Coutler (Brea, USA). Reagents for immunoglobulin A (IgA), immunoglobulin G (IgG), high-density lipoprotein (HDL), apolipoprotein A (ApoA), UTP were from Zybio (Zybio Company, Chongqing, China). High-concentration quality control (QC) materials for UTP was obtained from Bio-Rad Laboratories (Hercules, USA).

### Methods

This study was performed in June 2024 at Clinical Laboratory Center, Shaoxing People’s Hospital, Shaoxing, China. Before the experiment, AU5800 biochemistry analyzer was carefully set up and calibrated following the operating manual. The sample probes, the reagent probes, cuvettes, and mixbars were thoroughly cleaned and soaked. Pure water used had an electrical conductivity of ≤ 2 μs/cm.

### Evaluation of specific sample probe carryover in UTP testing

Five tubes of UTP were measured consecutively, with tubes 1-2 containing Bio-Rad high-concentration QC materials (designated as a1 and a2) and tubes 3-5 containing pure water (designated as b1, b2, and b3). This procedure was repeated on five consecutive days, and Equation 1 (Eq. 1) was used to calculate specific sample probe carryover (*13*).



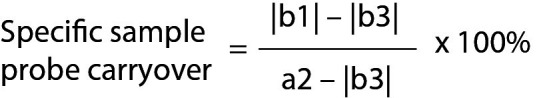



The average or medium carryover across five days was then determined. The specific sample probe carryover was expressed as mean ± standard deviation (SD) or median and inter-quartile range (IQR), depending on the data distribution. A carryover within the range of - 3% to 3% was considered indicative of no carryover (*14*).

### Evaluation of specific reagent probe and mixbars carryover in UTP testing

In our laboratory, the following eight reagents that are assigned to the inner cuvettes and the second analytical unit of the biochemical analyzer alongside UTP, were considered as potential items of carryover: ALP, Crea, HDL, CHE, IgA, IgG, ApoA and RF. These reagents were considered potential sources of carryover because they share the same reagent probe, mixbars, and cuvettes with UTP. Mixed 24-hour urine samples were used to evaluate the reagent probe and mixbars carryover in UTP testing. To address the potential for carryover from the mixbars to consistently affect subsequent measurements, including the second and even third ones, four samples of saline solution were added before evaluating the next potential carryover item to cleanse the sample probes and mixbars. The sequence of carryover evaluation, along with the samples and reagents used, is outlined in Table 1. Eight rounds of testing were conducted to evaluate each potential item of carryover.

**Table 1:** Sequence of specific reagent probe and mixbars carryover evaluation

**Sample order**	**Sample**	**Reagent**	**Code**	**Mixbar number**
1	Urine	Potential item of carryover	/	1
2	Urine	UTP	UTP1	2
3	Urine	UTP	UTP2	3
4	Urine	UTP	UTP3	1
5	Urine	UTP	UTP4	2
6	Saline	UTP	/	3
7	Saline	UTP	/	1
8	Saline	UTP	/	2
9	Saline	UTP	/	3
UTP - urinary total protein. / - no code required.				

The carryover was calculated using Eq. 2 and Eq. 3, with values below 5% considered indicative of no carryover (*14*).



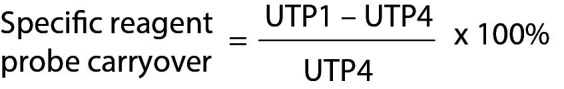





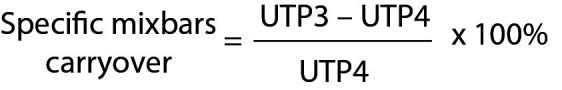



Since “potential items of carryover” shared three sets of mixbars, while the reagent probe was singular, UTP1 and UTP4 utilized the same set of mixbars. Prior to UTP1 testing, the reagent probe was immersed in the “potential item of carryover”, whereas prior to UTP4 testing, it was immersed in the UTP reagent and had been cleaned three times according to the instrument program. Moreover, the mixbars used in UTP4 were not exposed to reagents associated with carryover, making UTP4 an uncontaminated control for evaluating both the reagent probe and the mixbars. Accordingly, the ratio of (UTP1 - UTP4) / UTP4 was used to evaluate specific carryover from the reagent probe. The specific carryover of the mixbars was calculated as (UTP3 - UTP4) / UTP4 because UTP3 and the “potential item of carryover” shared the same set of mixbars.

### Evaluation of specific cuvettes carryover in UTP testing

In the cuvette reaction mixture, the reagent volume is typically tens to hundreds of times greater than the sample volume. Therefore, our evaluation of specific cuvette carryover focused on reagents assigned to the same cuvettes as UTP, including eight reagents: ALP, Crea, HDL, CHE, IgA, IgG, ApoA and RF. In addition, pure water was placed as a reagent in the reagent compartment of the second analytical unit, serving as control. Its position matched that of UTP and “potential items of carryover”, all of which were assigned to the inner cuvettes. According to the design of AU5800 biochemistry analyzer, each analytical unit is equipped with 408 cuvettes, divided into an inner cuvette ring and an outer cuvette ring, with each containing 204 cuvettes. Mixed 24-hour urine samples were prepared into 232 aliquots, each containing 100 µL. The experiment was conducted in two rounds.

22 samples were analyzed, with each sample tested using the aforementioned 9 reagents, resulting in a total of 198 test results. Additionally, 6 samples were analyzed exclusively for pure water reagent, generating 6 more test results. This resulted in a total of 204 tests, covering all 204 cuvettes in the inner ring. For each test, the analyzer allowed us to identify the specific cuvette used. We recorded both the reagent tested and its corresponding cuvette number for all 204 tests. In this round, the cuvettes were “contaminated” by the reagents tested.

The remaining 204 samples were all used for UTP testing, generating another 204 test results. For each of these tests, we identified the cuvette number used and traced it back to the reagent tested in the first round on the same cuvette. Since only 9 items were tested in the first round, each of these 9 items in the second round could correspond to multiple UTP data. The average or medium UTP value was calculated for each item and recorded as UTP(n), where n represented the item, *e.g.,* UTP(ALP) for alkaline phosphatase and UTP(H20) for the pure water control. The specific cuvette carryover was calculated using Eq. 4, with values below 5% considered indicative of no carryover.



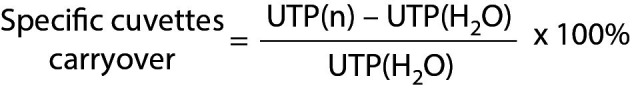



### Evaluation of cumulative sample carryover in UTP testing

We used mixed serum samples to simulate routine laboratory work, pure water was placed as a reagent in the reagent compartment of the second analytical unit’s inner ring. Water reagents were continuously tested, with mixed 24-hour urine interspersed as the sample. Urinary total protein concentrations were measured at intervals of 0 min, 10 min, 20 min, and 30 min. At each time point, UTP was tested three times to eliminate random errors. The average UTP value for each time point was calculated and recorded as UTP(time). The cumulative sample carryover was then calculated using Eq. 5. A range from - 3% to 3% was considered indicative of no carryover.



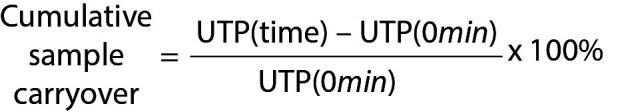



### Evaluation of cumulative reagent carryover in UTP testing

Pure water was used as sample and sequentially tested with eight reagents sharing the same analytical unit and inner cuvettes as UTP. Each reagent was tested continuously for 30 minutes, with mixed 24-hour urine interspersed as the sample. Urinary total protein concentrations were measured at 0, 10, 20, and 30 minutes. The cumulative sample carryover was calculated using Eq. 6, with values below 5% considered indicative of no carryover.



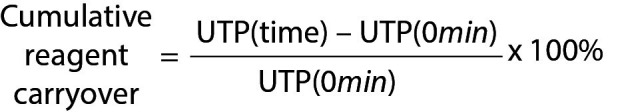



### Statistical analysis

All statistical analyses were done using SPSS version 26.0 (IBM Corp., Armonk, USA). The Shapiro-Wilk test was used for evaluation of data normality. Continuous variables with normal distribution were expressed as mean ± SD, and skewed data were expressed as median and IQR.

## Results

The average carryover of specific sample probes for UTP testing, tested over five days using AU5800 biochemistry analyzer, was - 0.11%, as shown in Table 2. The observed indicated that the carryover influence was negligible and did not compromise the accuracy of UTP measurement.

**Table 2:** Evaluation of specific sample probe carryover in UTP testing

**Day**	**UTP (g/L)**					**Specific sample probe carryover (%)**	**Average specific sample probe carryover (%)**
	**a1**	**a2**	**b1**	**b2**	**b3**		
1	0.551	0.543	- 0.004	0.004	- 0.006	- 0.37	
2	0.557	0.540	- 0.006	0.003	- 0.003	0.56	
3	0.556	0.548	- 0.005	0.000	- 0.006	- 0.18	- 0.11 ± 0.43
4	0.558	0.528	- 0.005	0.000	- 0.005	0.00	
5	0.550	0.544	- 0.002	0.001	- 0.005	- 0.56	
UTP - urinary total protein. a1 and a2 - tubes containing Bio-Rad high-concentration quality control materials. b1, b2 and b3 - tubes containing pure water. The average specific sample probe carryover is expressed as mean ± standard deviation. The average specific sample carryover within the range of - 3% to 3% was considered indicative of no carryover.							

As shown in Table 3, no specific carryover from the reagent probe or mixbars was observed during UTP testing. The carryover remained below 5%, well within acceptable limits.

**Table 3:** Evaluation of specific reagent probe and mixbars carryover in UTP testing

**Potential item of carryover**	**UTP1** **(g/L)**	**UTP2** **(g/L)**	**UTP3** **(g/L)**	**UTP4** **(g/L)**	**Specific reagent probe carryover (%)**	**Specific mixbars carryover (%)**
ALP	0.649	0.659	0.660	0.650	- 0.15	1.54
Crea	0.670	0.655	0.667	0.659	1.67	1.21
HDL	0.665	0.678	0.666	0.661	0.61	0.76
CHE	0.659	0.669	0.653	0.662	- 0.45	- 1.36
IgA	0.653	0.663	0.668	0.652	0.15	2.45
IgG	0.662	0.663	0.680	0.660	0.30	3.03
ApoA	0.658	0.656	0.665	0.656	0.30	1.37
RF	0.656	0.655	0.660	0.653	0.46	1.07
UTP - urinary total protein. ALP - alkaline phosphatase. Crea - creatinine. HDL - high-density lipoprotein. CHE - cholinesterase. IgA - immunoglobulin A. IgG - immunoglobulin G. ApoA - apolipoprotein A. RF - rheumatoid factor. The specific carryover from the reagent probe and mixbars with values below 5% was considered indicative of no carryover.						

The median concentration of UTP detected using water as the reagent was 0.671 g/L. The specific carryover for cuvettes associated with eight potential carryover items was below 5% and thus considered indicative of no carryover (Table 4).

**Table 4:** Evaluation of specific cuvettes carryover in UTP testing

**Potential item of carryover**	**UTP (g/L)**	**Specific cuvettes carryover (%)**
ALP	0.647 (0.639-0.653)	- 3.65
Crea	0.673 (0.667-0.680)	0.22
HDL	0.668 (0.660-0.673)	- 0.45
CHE	0.645 (0.635-0.651)	- 3.95
IgA	0.672 (0.663-0.678)	0.15
IgG	0.675 (0.668-0.681)	0.60
ApoA	0.654 (0.645-0.659)	- 2.61
RF	0.673 (0.666-0.682)	0.30
H20 (control)	0.671 (0.658-0.675)	NA
UTP - urinary total protein. IQR - interquartile range. ALP - alkaline phosphatase. Crea - creatinine. HDL - high-density lipoprotein. CHE - cholinesterase. IgA - immunoglobulin A. IgG - immunoglobulin G. ApoA - apolipoprotein A. RF - rheumatoid factor. The specific carryover from cuvettes with values below 5% was considered indicative of no carryover. Urinary total protein results are expressed as median and interquartile range. NA - not applicable.		

Despite the significantly higher protein concentrations in serum samples compared to urine samples, we found that the cumulative sample carryover at different time points was all within the range of - 3% to 3% (Table 5). This indicates that UTP measurement does not exhibit cumulative sample carryover.

**Table 5:** Evaluation of cumulative sample carryover in UTP testing

**Time**	**UTP (g/L)**	**Mean of UTP (g/L)**	**Cumulative sample carryover (%)**
	0.661		
0 min	0.681	0.668	NA
	0.661		
	0.667		
10 min	0.674	0.672	0.60
	0.674		
	0.679		
20 min	0.660	0.668	0.00
	0.665		
	0.681		
30 min	0.669	0.668	0.00
	0.655		
UTP - urinary total protein. The cumulative sample carryover ranging from - 3% to 3% was considered indicative of no carryover. NA - not applicable.			

The results showed that, except for the IgG reagent, the cumulative carryover for the other seven reagents was all below 5%, indicating that these seven reagents exhibited no cumulative carryover in UTP testing. In contrast, the IgG reagent exhibited significant cumulative carryover of 8.32%, 9.24%, and 10.32% at different time points, all exceeding the 5% threshold, as detailed in Table 6.

**Table 6:** Evaluation of cumulative reagent carryover in UTP testing

		**Time**											
**Potential item of carryover**		**0 min**			**10 min**			**20 min**			**30 min**		
	UTP (g/L)	0.674	0.667	0.665	0.669	0.673	0.663	0.659	0.644	0.657	0.658	0.664	0.667
ALP	Mean (g/L)	0.669			0.668			0.653			0.663		
	Carryover (%)	NA			- 0.15			- 2.39			- 0.90		
	UTP (g/L)	0.683	0.685	0.667	0.645	0.638	0.641	0.651	0.643	0.636	0.652	0.673	0.635
Crea	Mean (g/L)	0.678			0.641			0.643			0.653		
	Carryover (%)	NA			- 5.46			- 5.16			- 3.69		
	UTP (g/L)	0.630	0.637	0.624	0.643	0.650	0.627	0.647	0.646	0.664	0.638	0.627	0.628
HDL	Mean (g/L)	0.630			0.640			0.652			0.631		
	Carryover (%)	NA			1.59			3.49			0.16		
	UTP (g/L)	0.637	0.636	0.646	0.647	0.647	0.652	0.635	0.634	0.658	0.644	0.639	0.628
CHE	Mean (g/L)	0.640			0.649			0.642			0.637		
	Carryover (%)	NA			1.41			0.31			- 0.49		
	UTP (g/L)	0.642	0.651	0.644	0.666	0.643	0.645	0.629	0.642	0.641	0.647	0.652	0.647
ApoA	Mean (g/L)	0.646			0.651			0.637			0.649		
	Carryover (%)	NA			0.77			- 1.39			0.46		
	UTP (g/L)	0.628	0.642	0.634	0.647	0.644	0.629	0.643	0.648	0.648	0.651	0.648	0.644
IgA	Mean (g/L)	0.635			0.640			0.646			0.648		
	Carryover (%)	NA			0.79			1.73			2.05		
	UTP (g/L)	0.656	0.639	0.652	0.711	0.688	0.705	0.729	0.691	0.707	0.728	0.704	0.717
IgG	Mean (g/L)	0.649			0.703			0.709			0.716		
	Carryover (%)	NA			8.32			9.24			10.32		
	UTP (g/L)	0.636	0.642	0.645	0.647	0.647	0.640	0.629	0.649	0.639	0.635	0.638	0.640
RF	Mean (g/L)	0.641			0.645			0.639			0.638		
	Carryover (%)	NA			0.62			- 0.31			- 0.47		
UTP - urinary total protein. SD - standard deviation. ALP - alkaline phosphatase. Crea - creatinine. HDL - high-density lipoprotein. CHE - cholinesterase. IgA - immunoglobulin A. IgG - immunoglobulin G. ApoA - apolipoprotein A. RF - rheumatoid factor. The cumulative reagent carryover below 5% was considered indicative of no carryover. Mean - mean of three UTP tests results. NA - not applicable.													

**Figure 1:**
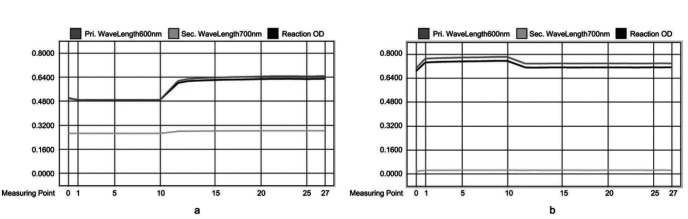
Reaction curves for UTP comparing contaminated and uncontaminated samples. a) The reaction curve of UTP testing with contamination. At measuring point 10, the reaction curve exhibited a significant rise, indicating contamination in UTP testing. b) The reaction curve of UTP testing without contamination. At point 10 the reaction curve showed a decline, suggesting that UTP testing was unaffected by contamination.

Subsequently, we examined the contaminated UTP reaction curve, which revealed noticeable differences compared to the previously uncontaminated UTP reaction curve (Figure 1). At the point of adding Reagent 2 (R2) and mixing the reaction mixture in the cuvette using mixbars (point 10), the reaction curve exhibited a significant rise (Figure 1a). This anomaly confirmed the presence of cumulative reagent residue on the mixbars, as the UTP reagent kit employs a single-reagent endpoint method that does not include R2. The shared use of a single set of mixbars for both IgG and UTP allowed the IgG reagent to be carried into the UTP reaction system, thereby causing carryover.

## Discussion

We demonstrated that cumulative reagent carryover from IgG reagent in UTP testing on AU5800 biochemistry analyzer, while no cumulative sample carryover or specific carryover was observed. We employ readily accessible laboratory materials for both samples and reagents, making it operational and eliminating the need for repetitive and unnecessary tests, thereby maximizing reagent cost savings. Our findings highlight the importance of evaluating UTP cumulative carryover, improving the accuracy and reliability of UTP testing.

The exploration of UTP carryover evaluation encompasses two aspects: specific carryover and cumulative carryover. Specific carryover was evaluated based on the location of contamination within the instrument, including essential components such as the sample probe, the reagent probe, mixbars, and cuvettes. In contrast, cumulative carryover was evaluated based on the accumulation of residual substances, further classified into cumulative sample carryover and cumulative reagent carryover. Given the variability in biochemical analyzers, the diversity of tests conducted, and the differing assay sequences across laboratories, most studies on carryover are confined to case analyses. After installing a new biochemical analyzer, Kyle’s laboratory observed negative total cholesterol values in several patient samples. Investigation revealed that significant reductions in total cholesterol were caused by carryover from creatine kinase reagents (*1*). Similarly, Kumari *et al.* identified an unusual scenario during routine testing, where lipase activities were elevated while amylase activities remained normal in some samples. Upon reanalyzing the lipase activities, they returned to normal, and it was determined that the interference originated from microbial lipase used in triglyceride reagents (*12*). Zhang *et al.* reported a case where a urine sample remained on the sample probe, leading to unusually elevated concentrations of serum potassium, urea nitrogen, and creatinine in subsequent serum specimens (*15*). These studies focus solely on specific carryover, lacking a systematic and comprehensive evaluation. The potential cumulative effect of carryover resulting from the increased number of assays and assay combinations performed by biochemical analyzers cannot be overlooked. Notably, they often fail to evaluate the cumulative effects of carryover, neglecting the potential risks posed by its accumulation over time. In our study, we evaluated the full range of possible carryover scenarios before introducing UTP as a clinical test in the laboratory. This proactive evaluation effectively mitigated the risk of adverse laboratory events caused by carryover.

The negative values detected in UTP testing data is likely due to the use of pure water for evaluating carryover. The reagent blank, which reflects the inherent absorbance of the reagent, is typically determined using pure water. Consequently, when pure water is used as the sample, the optical density value of the resulting reaction system may be almost equal to or even lower than that of the reagent blank.

In fact, the cumulative reagent carryover caused by IgG reagent, as discovered in this study, was traceable. Our laboratory uses immunoturbidimetry for IgG measurement. This method relies on the formation of immune complexes through antigen-antibody reactions (*16*). These complexes precipitate from the solution phase to form particles under the action of an aggregating agent (polyethylene glycol), resulting in turbidity in the reaction solution. Compared to other methodologies, the particles in immunoturbidimetry are more likely to remain in the subsequent reaction system (*3*). Residues will accumulate on the reagent probe, mixbars, and cuvettes, leading to contamination. Furthermore, the R2 reagent for IgG consists of 250 ml/L of sheep anti-human IgG antibodies, which are globulins capable of interacting with the UTP reagent kit. This cumulative reagent residue of globulins in R2 may be the primary factor contributing to the cumulative reagent carryover that affects UTP testing.

Cumulative reagent carryover, as described in this study, could be mitigated by implementing additional washing procedures, enhancing instrument maintenance, and optimizing reagent placement. However, it is important to note that introducing extra procedures significantly increases procedural complexity, extends processing time, and ultimately reduces testing efficiency.

There are several limitations to this study. Firstly, our evaluation of UTP carryover is specific to our laboratory conditions and is not a universally applicable method. We evaluated only eight reagents that share the same analytical unit and cuvettes with UTP, without conducting experimental validation for other reagents. Secondly, carryover may also be influenced by the instrument’s service life, including the cleanliness of pipelines, the wear of sample probes and mixbars. However, these factors were not evaluated in this study.

In conclusion, we conducted the full range of possible carryover scenarios in UTP testing using AU5800 biochemistry analyzer. Our findings revealed IgG reagents cause cumulative reagent carryover in UTP testing, while no cumulative sample carryover or specific carryover was observed. Our study highlights the necessity of thorough carryover evaluation in clinical chemistry analyzers, particularly in high throughput settings, to guarantee the dependability of laboratory results.

## Data Availability

The data generated and analyzed in the presented study are available from the corresponding author on request.

## References

[1] Kyle PB. Beware of carryover in modern chemistry analyzers. Clin Chem Lab Med. 2010;48:519–21. 10.1515/CCLM.2010.092 20148727

[2] Broughton PM. Carry-over in automatic analysers. J Automat Chem. 1984;6:94–5. 10.1155/S1463924684000201 18924602 PMC2547581

[3] Ling Q Peichang W Clinical Chemistry Task Force of Chinese Society of Laboratory Medicine. Expert consensus on the evaluation and treatment of the carryover of automatic biochemical analyzer. Chin J Lab Med. 2020;43:712–7.

[4] Haider MZ, Aslam A. Proteinuria. [Updated 2023 Sep 4]. In: StatPearls [Internet]. Available from: https://www.ncbi.nlm.nih.gov/books/NBK564390/. Accessed October 20th 2024.

[5] Currie G Delles C. Proteinuria and its relation to cardiovascular disease. Int J Nephrol Renovasc Dis. 2013;7:13–24. 10.2147/IJNRD.S40522 24379690 PMC3873205

[6] Coulter B. AU5800 Chemistry Analyzer Reference Manual. available from: https://www.beckmancoulter.com/download/file/wsr-224551/B81389AA?type=pdf. Accessed October 20th 2024.

[7] Lamb EJ MacKenzie F Stevens PE. How should proteinuria be detected and measured? Ann Clin Biochem. 2009;46:205–17. 10.1258/acb.2009.009007 19389884

[8] Orsonneau JL Douet P Massoubre C An improved pyrogallol red-molybdate method for determining total urinary protein. Clin Chem. 1989;35:2233–6. 10.1093/clinchem/35.11.2233 2582622

[9] Boneno MJ Fokakis M Armbruster D. Reagent carryover studies: Preventing analytical error with open clinical chemistry systems. Lab Med. 2005;36:705–10. 10.1309/MMLEYHNBY4WA12J6

[10] Karger AB Senn C Skogseth K Floodman S. Rare erroneous results on the Siemens Dimension Vista® platform due to urine carryover: A warning to current users. Clin Biochem. 2016;49:737–9. 10.1016/j.clinbiochem.2016.05.003 27174361

[11] Armbruster DA Alexander DB. Sample to sample carryover: a source of analytical laboratory error and its relevance to integrated clinical chemistry/immunoassay systems. Clin Chim Acta. 2006;373:37–43. 10.1016/j.cca.2006.04.022 16777083

[12] Kumari S Shekhar R Kumari R Study of analytical error in lipase assay. Ann Afr Med. 2023;22:55–60. 10.4103/aam.aam_135_21 36695223 PMC10064892

[13] Haeckel R. Recommendations for definition and determination of carry-over effects. J Automat Chem. 1988;10:181–3. 10.1155/S1463924688000380 18925212 PMC2547758

[14] Ling Qiu XC Liu Q Shen Y. Detection and handling of carryover of automatic biochemistry analyzer. J Med Res. 2007;36:64–7. [in Chinese]

[15] Zhang L Wang E Lu M. A case of abnormal results caused by urine contamination in serum during detection. Clin Lab. 2023;69:621–3. 10.7754/Clin.Lab.2022.220608 36912298

[16] Whicher JT Price CP Spencer K. Immunonephelometric and immunoturbidimetric assays for proteins. Crit Rev Clin Lab Sci. 1983;18:213–60. 10.3109/10408368209085072 6339164

